# Conventional Pig as Animal Model for Human Renal Drug Excretion Processes: Unravelling the Porcine Renal Function by Use of a Cocktail of Exogenous Markers

**DOI:** 10.3389/fphar.2020.00883

**Published:** 2020-06-12

**Authors:** Laura Dhondt, Siska Croubels, Peter De Paepe, Steven C. Wallis, Saurabh Pandey, Jason A. Roberts, Jeffrey Lipman, Pieter De Cock, Mathias Devreese

**Affiliations:** ^1^Department of Pharmacology, Toxicology and Biochemistry, Ghent University, Merelbeke, Belgium; ^2^Heymans Institute of Pharmacology, Ghent University, Ghent, Belgium; ^3^UQ Centre for Clinical Research, The University of Queensland, Royal Brisbane & Women's Hospital, Brisbane, QLD, Australia; ^4^Department of Intensive Care Medicine, Royal Brisbane & Women's Hospital, Brisbane, QLD, Australia; ^5^Centre for Translational Anti-Infective Pharmacodynamics, School of Pharmacy, The University of Queensland, Brisbane, QLD, Australia; ^6^Department of Pharmacy, Royal Brisbane & Women's Hospital, Brisbane, QLD, Australia; ^7^Division of Anaesthesiology Critical Care Emergency and Pain Medicine, Nîmes University Hospital, University of Montpellier, Nîmes, France; ^8^Department of Pharmacy, Ghent University Hospital, Ghent, Belgium; ^9^Department of Paediatric Intensive Care, Ghent University Hospital, Ghent, Belgium

**Keywords:** piglet, renal function, animal model, iohexol, para-aminohippuric acid, pindolol, fluconazole

## Abstract

Over recent years, pigs have been promoted as potential animal model due to their anatomical and physiological similarities with humans. However, information about the contribution of distinct renal elimination processes [glomerular filtration rate (GFR), effective renal plasma flow (ERPF), tubular secretion, and reabsorption] in pigs is currently limited. Therefore, a cocktail of renal markers, consisting of iohexol (GFR), para-aminohippuric acid (ERPF and net tubular anion secretion), pindolol (net tubular cation secretion), and fluconazole (net tubular reabsorption) was administered intravenously to 7-week-old male conventional pigs. Plasma and urinary concentrations were determined using validated analytical methods. The clearance of iohexol (GFR) was 97.87 ± 16.05 ml/min/m² (mean ± SD). The ERPF, calculated as the renal clearance of PAH, was 226.77 ± 62.45 ml/min/m², whereas the net tubular secretion of PAH was 130.28 ± 52.62 ml/min/m². The net tubular secretion of R-pindolol and S-pindolol was 13.53 ± 12.97 and 18.01 ± 39.23 ml/min/m², respectively. The net tubular reabsorption of fluconazole was 78.32 ± 13.52 ml/min/m². Overall, this cocktail of renal markers was considered to be safe for use in pigs since no adverse effects were observed. Iohexol, PAH and fluconazole were considered suitable renal marker to assess the porcine renal function. Pindolol seems less appropriate due to the high degree of nonrenal clearance in pigs. The values of GFR, ERPF, and anion secretion are within the same range for both human and pig. Regarding the tubular reabsorption of fluconazole, slightly higher values were obtained for pigs. Nevertheless, these results indicate the conventional pig could be an appropriate animal model to study renal drug elimination processes in humans.

## Introduction

The kidneys are involved in the excretion of various endogenous and exogenous substances. For drugs, which are frequently eliminated by the kidney, assessment of the renal function is important to enable development of robust doses that assure appropriate drug exposure. Generally, the net renal excretion is considered to be a combination of three major processes, namely glomerular filtration, tubular secretion, and tubular reabsorption. Currently, the assessment of the glomerular filtration rate (GFR) is considered the best overall measure of renal function. However, changes in each of the three renal processes can influence renal drug clearance (Verbeeck and Musuamba, 2009). Therefore, it is mandatory to characterize each specific renal elimination process separately. These three renal processes can be determined by use of several renal markers (Tett et al., 2003).

Since GFR assessment is the most widely used descriptor for renal function, a broad range of endogenous and exogenous GFR markers have been recognized (Stevens and Levey, 2009). The gold standard for GFR estimation is the measurement of inulin clearance (Stevens and Levey, 2009). However, this technique is not routinely applicable, since it requires constant infusion and timed urine collection (Gaspari et al., 1995). Therefore, the GFR is often estimated in daily practice using formulas derived from the serum creatinine concentrations. Iohexol, which is a nonradioactive GFR marker, is increasingly used in both veterinary and human medicine because its properties approach those of an ideal GFR marker (Miyamoto, 2001; Meucci et al., 2015; Zhang et al., 2017; Gaspari et al., 2018). More specifically, iohexol has only negligible binding to plasma proteins and is metabolically inert. Moreover, its administration is safe and urine collection is not mandatory (Delanaye et al., 2016). Iohexol is preferred above iothalamate as GFR marker since the latter may be affected by the existence of tubular secretion (Odlind et al., 1985). Due to its low cost and ease of handling, iohexol, as a marker of the GFR, has been repeatedly employed in swine (Frennby et al., 1997; Gasthuys et al., 2017a; Luis-Lima et al., 2018). Gasthuys et al. used iohexol to evaluate the maturation of the GFR in the growing conventional piglet at 8 days, 4 weeks, and 7 weeks of age (Gasthuys et al., 2017a). Luis-Lima et al. developed a simplified protocol to determine GFR using iohexol plasma clearance in swine (Luis-Lima et al., 2018). Frennby et al. administered iohexol to Swedish Landrace pigs to compare the iohexol clearance with ^51^Cr-EDTA and endogenous creatinine clearance (Frennby et al., 1997).

Much scarcer than markers for GFR, are validated markers for tubular secretion and reabsorption. Tubular secretion is a transporter mediated-process, which implies that its function is saturable and susceptible to competition. Separate carrier systems are present for anion and cation secretion (Vanginneken and Russel, 1989). Both para-aminohippuric acid (PAH) and probenecid have been utilized to characterize the anion transport system (Kinowski et al., 1995; Bonate et al., 1998). Besides being a marker for the anion secretion, PAH has also been extensively used to assess the renal plasma flow since it is freely filtered at the glomerulus, undergoes extensive tubular secretion and negligible reabsorption. It is almost completely cleared from the plasma as it passes through the kidney. However, the plasma concentration of PAH must be at an appropriately low level, otherwise saturation of the anion secretion occurs (Kinowski et al., 1995). To investigate the cationic secretion, both pindolol and famotidine have been used in humans (Hsyu and Giacomini, 1985; Dowling et al., 2001). Pindolol has previously been used as model compound for stereoselective renal clearance of organic cations, since it consists of an R- and S-isomer (Hsyu and Giacomini, 1985). In humans, fluconazole undergoes extensive tubular reabsorption, therefore it has been used as an indicator for the net tubular reabsorption (Debruyne and Ryckelynck, 1993; Gross et al., 2001; Tett et al., 2003; Udy et al., 2014).

To date, there remains a growing demand for appropriate animal models for the precise evaluation of the efficacy and safety of therapeutic drugs (Dziegiel et al., 2018). Dogs and monkeys have been used as the nonrodent species of choice in preclinical pharmaceutical drug research. However, the interest has grown to use swine as a translational animal model in biomedical research due to their high degree of anatomical and physiological similarities with humans (Swindle et al., 2012). With respect to the kidney, the structure, function, and physiology of the mature porcine kidney are postulated to be comparable to that of humans, making pigs a potentially suited model for studying human renal drug excretion processes (Dalmose et al., 2000; Gasthuys et al., 2016).

Previously, Gasthuys et al. demonstrated that the maturation of the GFR, determined as iohexol plasma clearance, was comparable between children and growing pigs, making the growing pig a potential good preclinical model for pediatric drug research and an amenable model to study renal (patho)physiology (Gasthuys et al., 2017a). To date, GFR estimation in pigs is frequently described in literature; however, limited information on the other porcine renal excretion processes is available. Nevertheless, this information could contribute to the evaluation of the suitability of the pigs as animal model.

A single cocktail approach, in which a serie of marker compounds is administered at once followed by repetitive blood and urine sampling, has been validated in humans (Gross et al., 2001; Udy et al., 2014). This cocktail consisted of sinistrin to determine the GFR, PAH to measure the effective renal plasma flow (ERPF) and net tubular anion secretion, pindolol to evaluate the net tubular cation secretion, and fluconazole as an indicator of the passive reabsorption. To the authors' knowledge, a single cocktail approach to elucidate the renal function in pigs has never been applied. Therefore, the aim of this study was to assess the feasibility and validity of administering a cocktail of renal markers to pigs in order to characterize the renal excretion processes in 7-week-old pigs. This cocktail consisted of (1) iohexol to measure the GFR, (2) PAH to evaluate ERPF and net tubular anion secretion, (3) pindolol to evaluate net tubular cation secretion, and (4) fluconazole as an indicator of tubular reabsorption. To investigate the appropriateness of the swine as a potential translational animal model, the results of this study were compared with human data.

## Materials and Methods

### Animals

Eight healthy, stress resistant, 6-week-old male piglets (Landrace × Large White × Maximus, Seghers Hybrid^®^, Wuustwezel, Belgium) were recruited for this study. Upon arrival piglets were group-housed in standard pig stables (2.30 × 2.40 m) with *ad libitum* access to water and feed (Piggistart Opti^®^, Aveve, Leuven, Belgium). During the whole experimental period, stables were enriched with rubber toys, balls of different size, and cotton towels. After a 5-day acclimatization period, a double-lumen jugular catheter was inserted following the procedure described by Gasthuys et al., permitting accurate intravenous (IV) administration of the renal markers and blood collection (Gasthuys et al., 2017b). During anesthesia, a human stoma ring (Esteem synergy^®^ Uro, 48 mm, ConvaTec, Belgium) was attached around the prepuce of the piglets to allow urine collection (Gasthuys et al., 2017b). After surgery, the pigs were housed individually to avoid displacements of the catheters and stoma rings. Catheters were flushed at least twice daily with heparinized 0.9% NaCl (50 IU/ml), and the bandages were changed daily.

The study was conducted with consent of the Ethical Committee of the Faculty of Veterinary Medicine and the Faculty of Bioscience Engineering of Ghent University (EC 2017/24). Care and use of animals were in compliance with the Belgian and European legislation on animal welfare and ethics (European Parliament and Council of the European Union, 2010; Flemish Government, 2017).

### Experimental Design

After a one day recovery period, urine pouches were attached to the stoma ring just before administration of the drugs. The pigs (7 weeks old, weighing 9.75 ± 1.61 kg) received the following renal markers as separate single IV boluses using the proximal lumen of the jugular catheter: iohexol (64.7 mg/kg BW, Omnipaque^®^ 300, GE Healthcare, Belgium), PAH (10 mg/kg BW), pindolol (0.05 mg/kg BW), and fluconazole (0.5 mg/kg BW, Diflucan^®^ 200 mg/100 ml). The commercial available powders of pindolol and PAH sodium salt, both purchased from Sigma-Aldrich (Bornem, Belgium), were dissolved separately in sterile, isotonic saline 0.9% solution prior to administration at a concentration of 0.8 mg/ml and 100 mg/ml, respectively. Due to the poor solubility of pindolol in water, the solution was slightly acidified with glacial acetic acid (0.04 v/v%) followed by sonication to enhance solubility (Fornal et al., 1999). The administered doses were determined based on available literature and practical considerations (Friedli et al., 1986; Gross et al., 2001; Gasthuys et al., 2017a). In the study of Gross et al., fluconazole and pindolol were both administered orally to humans (Gross et al., 2001). However, to minimize the risk of confounding factors, it was decided to administer both compounds IV and in lower doses than when given orally to humans. A dose of 0.5 mg/kg fluconazole (Diflucan^®^ 200 mg/100 ml) ensured a limited volume could be administered (<5 ml) as a bolus injection. Blood was sampled *via* the other lumen of the catheter at 0, 5, 15, 30, 45, and 60 min, and 2, 4, 6, 8, 12, 24, 36, 48, and 72 h post administration and collected into K_3_EDTA collection tubes (Vacutest^®^, Piove die Sacco, Kima, Italy). The samples were kept on ice and centrifuged (2,095 × *g*, 10 min, 4°C) within 2 h. Multiple timed urine collections were performed over a 48 h time period. The total volume of urine voided in each time period was registered. Aliquots of plasma were stored at ≤−80°C until analysis. Urine samples were initially stored at −20°C for 6 weeks, but were subsequently stored at −80°C for further storage.

### Quantification of the Renal Markers

Total plasma iohexol and PAH concentrations were quantified simultaneously using a validated ultrahigh performance liquid chromatography tandem mass spectrometry (UHPLC-MS/MS) method as previously described by Dhondt et al. (2019) (Dhondt et al., 2019). The lower limit of quantification (LLOQ) was 0.25 µg/ml for both compounds. The same UHPLC method was used for analysis of PAH in urine samples with slight modifications. The LLOQ was 0.25 µg/ml. A brief description of this latter method, including validation results, is presented in the supplement. Concentrations of R-pindolol, S-pindolol, and fluconazole in plasma and urine were determined using UHPLC-MS/MS. The acceptance criteria described in the FDA guideline were used for the validation of the pindolol and fluconazole methods [34]. The LLOQs were 0.2 ng/ml for R-and S-pindolol in both urine and plasma. For fluconazole, the LLOQ was 0.1 µg/ml in plasma and urine. A brief description of these methods and validation results are given as supplementary material.

To determine the ratio of R- and S-pindolol in the administered powder, a standard solution of 0.5 ng/ml in methanol:water (50:50, v/v), using the same powder as administered to the pigs, was made and analyzed together with the samples.

### Plasma Protein Binding

Plasma protein binding of PAH, pindolol, and fluconazole was determined using an *in vitro* approach. Fresh blank pig plasma was spiked with a standard aqueous solution of the compound at three concentration levels: 0.50, 5.0 and 20 µg/ml for PAH, 0.1, 0.5, and 1 µg/ml for fluconazole and 5, 25 and 50 ng/ml for pindolol. Three aliquots of each concentration level were analyzed the same way as the pharmacokinetic (PK) study samples as described above to determine the total plasma concentration. Three other aliquots of each concentration were incubated for 1 h in a hot water bath of 39°C to replicate the pig's core body temperature and subsequently transferred onto an ultrafiltration device. An Amicon^®^ Ultra-0.5 ultrafiltration device (30 kDa; Merck, Overijse, Belgium) was used in the case of PAH and fluconazole, and centrifugation occurred at 4,000 × *g* for 10 min at 39°C. In the case of pindolol, a Microcon^®^ Ultracel YM-30 (Millipore Corporation, Bedford, USA) was used and centrifuged (16,000 × *g*, 15 min, 39°C). The volume of ultrafiltrate had to be below 25% of the total volume applied on the ultrafiltration device. Thereafter, the obtained filtrate was analyzed in the same way as the PK study samples to determine the unbound plasma concentration. The following equation was used to determine the unbound fraction in plasma:

$$f_{u}=\frac{C_{unbound}}{C_{total}}$$

Nonspecific binding (NSB) of compounds to the ultrafiltration devices can influence to a large extent the results (Lee et al., 2003). NSB binding to the filter was determined by adding a standard solution in phosphate buffered saline (PBS) of the respective compound on the filter. The duration of centrifugation was reduced to 2.5 and 1.0 min for Microcon^®^ and Amicon^®^ filters, respectively. In that way, the ultrafiltrate volume remained below 25% of the total volume applied. If necessary, the concentration obtained after filtration was corrected for NSB using the following formula:

$$C_{unbound,corrected}=\frac{C_{unbound,measured}}{1-NSB}$$

Where NSB is calculated as

$$NSB=\frac{C_{PBS,NF}-C_{PBS,F}}{C_{PBS,NF}}$$

where C_PBS,NF_ is the drug concentration in nonfiltered PBS and C_PBS,F_ the drug concentration in the PBS filtrate after centrifugation. Results are presented as mean ± SD.

### Pharmacokinetic and Statistical Analysis

Pharmacokinetic modeling of the plasma concentration–time data was performed using Phoenix^®^ 8.1 (Certara, Cary, NC, USA). Values below the LOQ were excluded from the dataset. The structural model for both iohexol and fluconazole was a two-compartmental model with first order elimination. A multiplicative error model was used. For both PAH, R-and S-pindolol, a one-compartmental model with first order elimination and multiplicative error model was used. The estimated primary parameters were volume of distribution (Vd) and total body clearance (CL_TOT_). Also the following secondary parameters were calculated: elimination half-life (T_1/2el_), elimination rate constant (K_e_), volume of distribution at steady state (V_ss_), and the area under the curve from time 0 h to infinity (AUC_0→inf_).

The clearances of the renal markers were normalized and indexed to BW (ml/min/kg) and body surface area (BSA, ml/min/m²) using the Meeh equation ($BSA\left(dm^{2}\right)=9*BW{\left(kg\right)}^{2/3}$) (Gasthuys, 2017).

The cumulative amount of unchanged compound recovered in the urine (A_e_) was calculated taking the sum of the amount excreted at every collection point. This amount was calculated by multiplying the observed concentration by the volume collected at every collection point. Subsequently, renal clearances (CL_R_) of the pindolol isomers and PAH were calculated by

$$CL_{R}=\frac{A_{e}}{AUC_{0\to inf}}$$

where AUC_0→inf_ is the area under the plasma concentration–time curve extrapolated to infinity. Since for fluconazole urine sampling was only performed up to 48 h after administration, A_e_, collected over 48 h, was divided by the AUC_0→48h_ to obtain the CL_R_. The AUC_0→48h_ was calculated by noncompartmental analysis (NCA) using the linear up-log down trapezoidal method. The nonrenal clearance (CL_NR_) was obtained by subtracting the CL_R_ from CL_TOT._ The CL_TOT_ of iohexol is a measure for the GFR. The CL_R_ of PAH is used to estimate the ERPF. The filtration fraction (FF) was calculated by

$$FF\left(\%\right)=\frac{GFR}{ERPF}*100$$

The filtration clearance of the unbound marker (CL_fil_) was calculated by f_u_ × GFR, where f_u_ is the unbound fraction of the compound in plasma. Net tubular anion and cation secretions were calculated as CL_R_ − CL_fil_. CL_fil_ − CL_R_ of fluconazole was used to calculate the net tubular reabsorption of fluconazole. To investigate if it was possible to reduce the time span of urine and blood collection the PK calculations of fluconazole were repeated using the collected data up to 24 h. The urinary recovery, which is the fraction of the administered dose recovered in the urine was calculated as A_e_/D. Results are presented as mean ± standard deviation (SD).

Differences between the PK parameters for the pindolol isomers were investigated using a Wilcoxon signed rank test [SPSS 25.0 (IBM, Chicago, IL, United States)]. The same approach was used to assess the agreement between the CL_R_ fluconazole after calculation using the data up to 24 and 48 h. The significance level was set at p = 0.05.

### Comparison With Human Values

To assess the suitability of the pig as animal model, the porcine clearance values were compared with human adult values in literature. Pigs within the 4–14 week age category correspond with a human age of 2–12 years (Gasthuys et al., 2016). It is postulated that human adult values for GFR, ERPF, and anion secretion are obtained at an age of 2 years (Rubin et al., 1949). In pigs, adult values of GFR and ERPF are reached around an age of 8 weeks. Adults values for the extraction of PAH, a measure for tubular secretion, are obtained at 3 weeks of age (Friis, 1979). Therefore, it seemed permissible to compare the porcine values of the distinct renal clearance processes in this study with human adult values reported by Gross et al. (Rubin et al., 1949; Gross et al., 2001). The cocktail used in the study of Gross et al. was similar to that administered in the presented study. It consisted of sinistrin, PAH, pindolol, and fluconazole. The latter two compounds were, in contrast to this study, administered orally. Gross et al. determined PK parameter values in the humans using noncompartmental analysis. Since Gross et al. only reported PK values not corrected for BSA and BW, the values presented in their study were corrected for the mean BW, which was 72 kg (Gross et al., 2001). The mean BSA was estimated using the formula of Dubois, where the mean BW was 72 kg and mean height 178 cm.

## Results

All piglets survived the surgical procedure without any complication. After administration of the cocktail of renal markers no adverse effects were observed. The pigs showed a normal activity and appetite. For two pigs, leakages of the urine bags were observed during the 48 h urine collection period. Therefore, these pigs were excluded in the calculation of the renal clearances (**Tables 1** and **2**). Those PK parameters, which were independent of urine collection, all pigs were included (**Table 3**). During the 48 h observation period, the urinary flow rate was 2.03 ± 1.00 ml/kg/h.

**Table 1:** Main pharmacokinetic parameters (mean ± SD) of iohexol (64.7 mg/kg BW), PAH (10 mg/kg BW), pindolol (0.05 mg/kg BW), and fluconazole (0.5 mg/kg BW) after intravenous bolus administration to 7-week-old male pigs.

	Iohexol (n = 8)	PAH (n = 6)	S-pindolol (n = 6)	R-pindolol (n = 6)	Fluconazole (n = 6)
AUC_0→inf_ (µg * h/ml)	265.19 ± 32.40	5.43 ± 1.04	0.0028 ± 0.0010^a^	0.0082 ± 0.0028^a^	18.73 ± 1.95
A_e_ (µg)^1^	/	29,512 ± 5751	5.34 ± 2.87^b^	6.30 ± 3.05^b^	2,548 ± 543^1^
CL_TOT_ (mL/min/kg)	4.12 ± 0.54	31.53 ± 5.38	161.26 ± 62.79^c^	56.85 ± 17.46^c^	0.45 ± 0.049
CL_R_ (mL/min/kg)	/	9.51 ± 2.44	3.28 ± 1.54^d^	1.34 ± 0.53^d^	0.32 ± 0.05
CL_NR_ (mL/min/kg)	/	22.02 ± 3.76	157.98 ± 62.42^e^	55.51 ± 17.34^e^	0.13 ± 0.039
Urinary recovery (%)	/	30.09 ± 5.06	2.18 ± 1.01^f^	2.49 ± 1.04^f^	/

^a−e^Significant differences (p < 0.05) between PK parameters of the pindolol isomers are indicated with the same alphabetical character superscript.

AUC_0→inf_, Area under the plasma concentration–time profile extrapolated to infinity, A_e_, the cumulative amount of unchanged compound recovered in the urine; CL_TOT_, total body clearance; CL_R_, renal clearance; CL_NR_, non-renal clearance.

^1^Total amount observed in urine collected over 48 h.

**Table 2:** Clearance values (mean ± SD) of the individual renal markers (iohexol (IOH), para-aminohippuric acid (PAH), pindolol (PIND), and fluconazole (FLUC)) in healthy human adults and 7-week-old male pigs.

	PIG		HUMAN^1^	
	ml/min/m²	ml/min/kg	ml/min/m²	ml/min/kg
GFR = CL_TOT_ IOH	97.87 ± 16.05	4.12 ± 0.54	68.78 ± 21.16^2^	1.81 ± 0.56^2^
ERPF = CL_R_ PAH	226.77 ± 62.45	9.51 ± 2.44	247.09 ± 77.25	6.48 ± 2.02
Net secretion PAH = CL_SECR_ PAH	130.28 ± 52.62	5.47 ± 2.13	189.95 ± 70.90	4.99 ± 1.86
Net tubular reabsorption FLUC = CL_REA_ FLUC	78.32 ± 13.52	3.28 ± 0.43	49.21 ± 15.34	1.29 ± 0.40
Net tubular secretion R-PIND = CL_SECR_ R-PIND	13.53 ± 12.97	0.57 ± 0.54	69.84 ± 23.81	1.83 ± 0.63
Net tubular secretion S-PIND: CL_SECR_ S-PIND	18.01 ± 39.23	0.77 ± 1.64	80.42 ± 37.04	2.11 ± 0.97

GFR, glomerular filtration rate; CL_TOT_, total body clearance; CL_R_, renal clearance; CL_SECR_, clearance by secretion; CL_REA_, reabsorption at the level of the kidney.

^1^Human, adult values were calculated from the data published by Gross et al. (2001). ^2^The GFR in humans was calculated as the renal clearance of sinistrin.

**Table 3:** Overview of the pharmacokinetic (PK) parameters of the pindolol isomers (0.05 mg/kg BW) and fluconazole (0.5 mg/kg) after IV administration to 7-week-old pigs.

	S-pindolol (n = 8)	R-pindolol (n = 8)	Fluconazole (n = 8)
V_ss_ (L/kg)	6.60 ± 1.69^1^	2.12 ± 0.56^1^	0.98 ± 0.04
CL_TOT_ (ml/min/kg)	173.08 ± 58.48^2^	54.20 ± 16.00^2^	0.45 ± 0.05
K_e_ (1/h)	1.58 ± 0.32	1.54 ± 0.26	0.03 ± 0.00
T_1/2el_(h)	0.46 ± 0.12	0.46 ± 0.07	25.71 ± 2.75
F_u_	0.62 ± 0.00^3^	0.19 ± 0.03^3^	0.89 ± 0.07

^1−3^Significant differences (p < 0.05) between PK parameters of the pindolol isomers are indicated with the same numerical character superscript.

V_ss_, volume of distribution at steady state; CL_TOT_, total body clearance; K_e_, elimination rate constant; T_1/2el_, elimination half-life; F_u_, free, non-protein bound, fraction.

**Figure 1:**
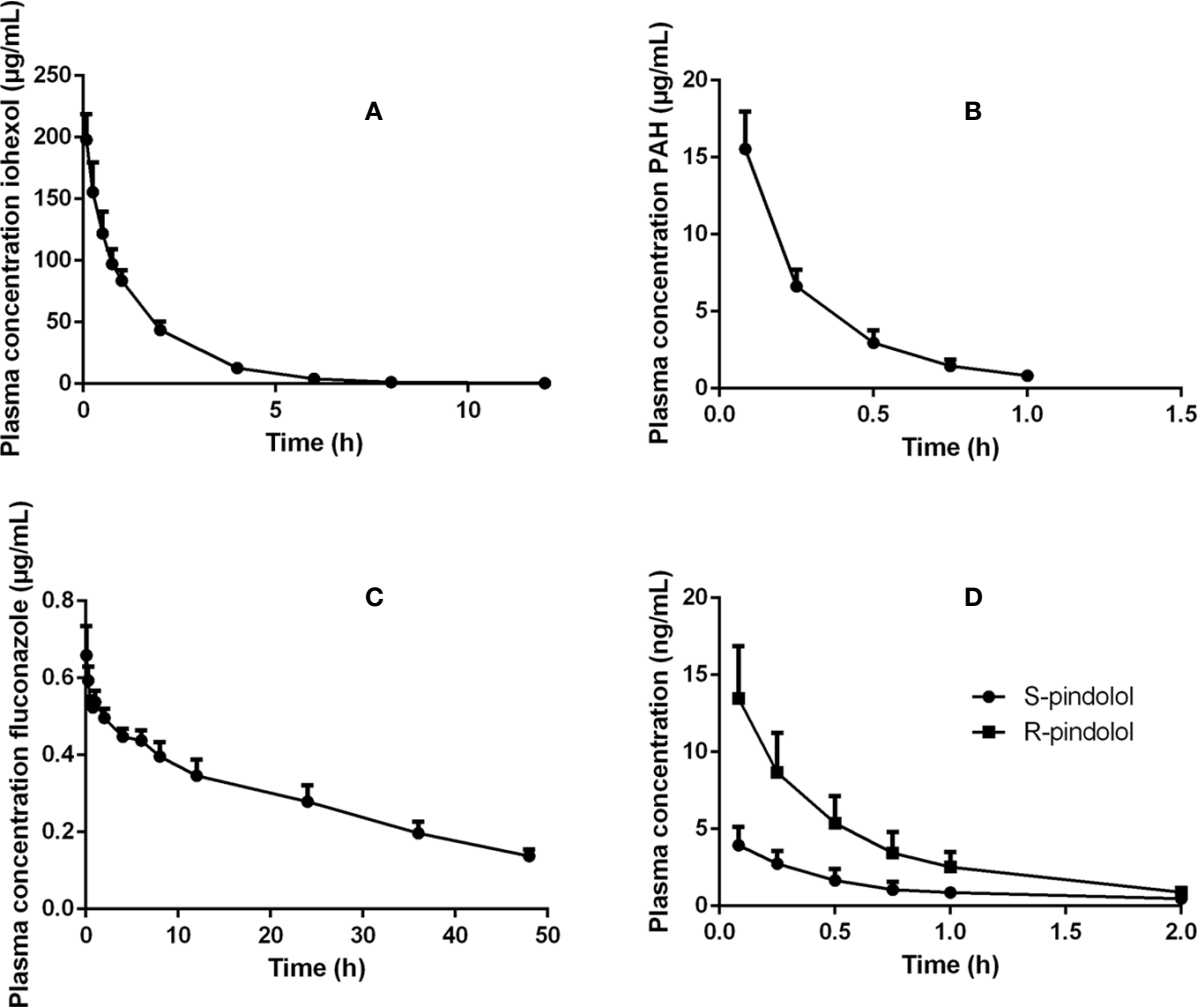
Plasma concentration–time profiles of **(A)** iohexol (64.7 mg/kg BW), **(B)** PAH (10 mg/kg BW), **(C)** fluconazole (0.5 mg/kg BW), and **(D)** pindolol (0.05 mg/kg BW) after intravenous bolus administration of these renal markers to 7-week-old male pigs.

Mean plasma concentration–time profiles (+SD) for the different renal markers are presented in **Figure 1**. **Table 1** gives an overview of the values of the PK parameters, which were used to calculate renal clearances of the administered renal markers.

The *in vitro* plasma protein binding experiment showed that PAH was not bound to plasma proteins (−4.4 ± 2.2%), resulting in an f_u_ of 1.0 for PAH. Since no protein binding of PAH was observed, it was not necessary to determine the NSB of this compound to the filter. Only 30.09 ± 5.06% of the total PAH dose administered to the pigs was recovered in the urine, indicating nonrenal clearance contributes to a large extent to the elimination of PAH. The effective renal plasma flow, calculated as renal clearance of PAH, was 9.51 ± 2.44 ml/min/kg. The GFR, measured as the total clearance of iohexol, was 4.12 ± 0.54 ml/min/kg. The mean FF was 44.15 ± 8.57%.

After IV administration of pindolol, containing 50.98 ± 0.66% R-pindolol and 49.02 ± 0.66% S-pindolol, stereoselective PK was observed. The urinary recovery was significantly higher for R-pindolol than S-pindolol (p < 0.05). Both the renal (p < 0.05) and nonrenal (p < 0.05) as well as the total clearance (p < 0.05) were higher for S- than R-pindolol. This suggests that both metabolism and renal excretion occur stereoselectively. This could be partially attributed to the lower plasma protein binding of S-pindolol (f_u_ = 0.62 ± 0.00) in contrast to R-pindolol (f_u_ = 0.19 ± 0.03). The difference in plasma protein binding is also reflected in a significantly higher V_ss_ of S-pindolol compared to R-pindolol (p < 0.05) (**Table 3**). No statistically significant differences were observed in the elimination rate constant (p = 0.67) and elimination half-life (p = 0.78). The PK parameters of pindolol, used to calculate the extent of cation secretion, are presented in **Table 1**. In **Table 3**, additional PK parameters of the pindolol isomers are presented.

In contrast to pindolol, where no significant NSB to the Microcon filter was observed, a NSB of ±20% for fluconazole was observed for both tested filters (Amicon and Microcon). After correction for NSB, a f_u_ of fluconazole of 0.89 ± 0.07 was obtained. The renal clearance of fluconazole calculated over a time period of 24 h (0.33 ± 0.06 ml/kg/min) did not differ statistically (p = 0.25) from the renal clearance estimated over 48 h (0.32 ± 0.05 ml/kg/min), indicating that renal clearance can be reliably estimated over a time period 24 h after dosing. The pharmacokinetic parameters of fluconazole are presented in **Tables 1** and **3**.

An overview of adult human and 7-week-old porcine values of GFR, ERPF, anion secretion, cation secretion, and net tubular reabsorption is presented in **Table 2**.

## Discussion

The present study offers new insights in the different porcine renal excretion processes by use of a cocktail of renal markers without the possible confounding effect of anesthesia (Deutsch, 1975). The pigs displayed no adverse effects during the study, indicating that the concomitant administration of these renal markers was safe in pigs. Since an optimal noninvasive urine collection technique for female piglets is currently lacking, only male pigs were included in this study (Gasthuys et al., 2017b). The major concern during the animal trials was the feasibility of urine collection using urine pouches over 48 h in nonsedated and nonrestricted pigs. Nevertheless, only two pigs showed an isolated event of leakage. The use of metabolic cages would circumvent the use of urine bags for urine collection; however, in the latter case the pigs are restricted in freedom of movement.

As for human adults and infants, quite some variability was observed in urinary output of the individual pigs, which is partially attributed to individual differences in water intake (Mattsson and Lindström, 1995). Generally, the normal urine output of a child is considered to be within the range of 1–2 ml/kg/h, which is in the same range as observed in the studied pigs (2.03 ± 1.00 ml/kg/h) (Hazinski, 1992).

**Figure 2:**
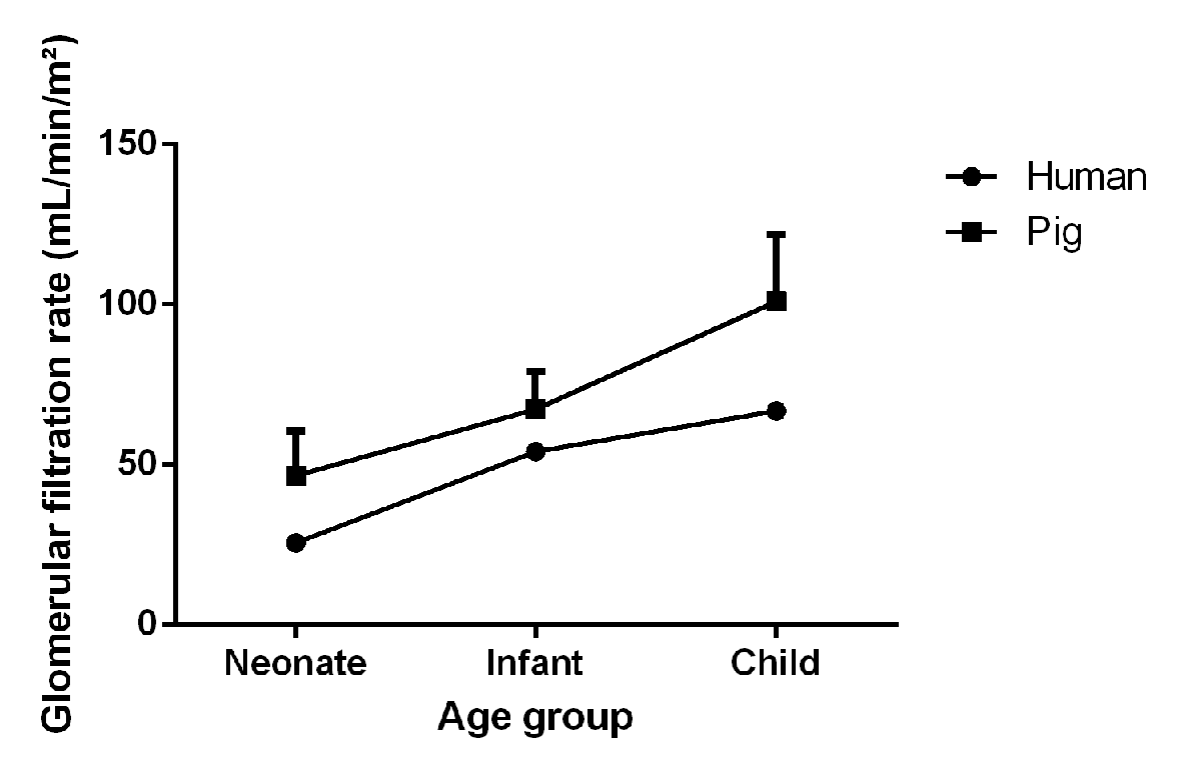
Visual representation of the maturation of the glomerular filtration rate (mean + SD) in male, conventional pigs and humans at different age categories (neonate, infant, and child). Porcine and human data were adopted from Gasthuys et al. and Schwartz et al. respectively (Schwartz and Furth, 2007; Gasthuys et al., 2017a).

The clearance of iohexol (97.87 ± 16.05 ml/min/m²) of these 7-week-old pigs was similar to the iohexol clearance reported by *Gasthuys et al*. in pigs of 7 weeks of age, namely 100.92 ± 20.84 ml/min/m² (Gasthuys et al., 2017a). When comparing the GFR to human values, taking into consideration the age correlation between humans and pigs proposed by Gad et al. the values obtained in humans (range 63–75 ml/min/m²) are approximately 70% to those of pigs (Gad, 2007; Schwartz and Furth, 2007). Interestingly, in each corresponding age category from neonate, infant to child, the human value is between 55 and 80% of the value obtained in pigs. Although the absolute GFR values were lower in case of humans, a similar trend in maturation between humans and pigs could be observed (**Figure 2**).

Since PAH is primarily excreted by the kidney, it has been widely used for the assessment of the ERPF in humans. Generally, PAH is administered as an IV constant rate infusion whereafter the ERPF is estimated as the renal clearance of PAH. PAH administration as a single bolus injection has also been performed in humans and dogs (Hirata-Dulas et al., 1994; Laroute et al., 1999). However, there has been some criticism about this latter approach, because of the differences in observed clearances between the bolus and infusion method (Hirata-Dulas et al., 1994; Tett et al., 2003). Nevertheless, these differences in clearance did not reach statistical significance in humans. To minimize the chance of saturating secretion, PAH doses for renal function assessment by bolus injections should result in plasma concentrations below those that saturate transporters. In humans, saturation of the transport system is observed at a concentration of 300–600 µg/ml (Tett et al., 2003). In this study, PAH plasma concentrations below 30 µg/ml were reached, as can be seen in **Figure 1**, minimizing the risk of saturation of the tubular secretion of PAH. Furthermore, the maximal PAH plasma concentrations obtained in this study are within the concentration ranges obtained after continuous infusion or subcutaneous administration of PAH during renal function studies in pigs (Friis, 1979; Link et al., 1985; Willis et al., 1997). Remarkable is the high nonrenal plasma PAH clearance (22.02 ml/min/kg) observed in pigs in contrast to that of adult humans (2.72 ml/min/kg) (Gross et al., 2001). In pigs, nonrenal elimination contributes for approximately 70% of the total clearance of PAH, which highlights probably the high acetylation capacity of the pig, as reported in literature (Cunningham et al., 2010). Also in humans, N-acetyl PAH is the major metabolite. The nonrenal clearance accounts for approximately 15 to 30% of the total drug elimination of PAH in this species (Prescott et al., 1993). Interesting to notice is also the presence of genetic polymorphism in N-acetyltransferase, resulting in slow and fast acetylators in humans (Walker et al., 2009). In dogs, no acetylated metabolites were observed (Laroute et al., 1999). One report suggests that PAH cannot be used to determine the ERPF of pigs due to the presence of acetylation of PAH at the level of the kidney (Nielsen et al., 1966). However, Gyrd-Hansen and Rasmussen (1970) demonstrated *in vivo* that the amount of PAH acetylated by the porcine kidney varied between −21.1 and 10.1%, with a mean of −0.1%, indicating that besides acetylation also deacetylation can occur in the kidney. This author suggests that when the number of animals is small, total PAH (PAH + N-acetyl PAH) determination is preferable, whereas when a larger number of animals are used the mean clearance of total PAH (PAH + N-acetyl PAH) and PAH will give identical values since the average amount of PAH acetylated by the porcine kidney is −0.1% (Gyrd-Hansen and Rasmussen, 1970). Since this study was conducted with eight animals, it was assumed that the mean acetylation was on average 0%, making the mean renal clearance of PAH a good estimator of the mean ERPF in pigs. The renal clearance of PAH corrected for BSA was comparable between human and pigs (247.09 *vs* 226.77 ml/min/m²). When indexing to BW a higher value was obtained for pigs (9.51 ml/min/kg) in contrast to humans (6.48 ml/min/kg). For the net tubular secretion of PAH, human and porcine values were quite comparable when indexed for BW (4.99 *vs* 5.47 ml/min/kg) and BSA (189.95 *vs* 130.28 ml/min/m²). The filtration fraction observed in pigs (44.1%) was higher than that observed in humans (20–30%), but was in accordance with the values observed in pigs by Friis *et al*. (Friis, 1979; Wainer et al., 1980; Gross et al., 2001; Udy et al., 2014). It is important to notice that the FF calculated by Friis *et al*. was performed in a different way than in this study and the referred human studies. To calculate the FF, Friis took the extraction of PAH (E_PAH_) by the kidney into account, as seen in the formula (FF = GFR/(CL_R__PAH_/E_PAH_)). When applying this formula, he obtained a mean FF of 33 ± 5% in piglets aged 1-79 days. Calculation of the FF, following the same formula as in this study, yielded a FF of 40 ± 6%, which is very similar as in the presented study (Friis, 1979).

In contrast to the study of Gross et al., in which fluconazole and pindolol were given orally, the piglets received in this study an IV bolus of both compounds due to practical considerations and the absence of oral bioavailability as confounding factor (Gross et al., 2001). One literature report described a different effect of pindolol on the renal function when given IV or orally in hypertensive humans (Wainer et al., 1980). A significant decline in GFR against baseline was observed after IV pindolol administration to hypertensive patients, whereas no significant effect was detected after oral administration. However, the decrease in GFR in case of IV administration was on average 4.37% (range: 0.96−9.43%), which is rather of less clinical relevance. In dogs, IV administration of pindolol produced slight but insignificant decrements in ERPF and GFR (Epstein et al., 1985). Taking these considerations into account, it seemed acceptable to use IV administration. Furthermore, the IV dose of 0.05 mg/kg that was used in the present study resulted in much lower plasma concentrations than an oral dosage of 5 or 15 mg of pindolol in humans, minimizing the risk of potential adverse renal effects (Gross et al., 2001; Udy et al., 2014). Furthermore, in pigs, in whom mycocardial infarction was induced, an equivalent IV dose of 0.05 mg/kg pindolol did not induce any hemodynamical changes (Friedli et al., 1986). To the authors' knowledge, information about the PK of pindolol in pigs is not available. In contrast to humans (f_u_ = 0.45), stereoselective binding of pindolol to plasma proteins was observed, with f_u_ of 0.19 and 0.62 for R-and S-pindolol, respectively (Hsyu and Giacomini, 1985). This stereoselective binding is also reflected in the stereoselective V_ss_ values. The V_ss_ of S-pindolol (6.60 L/kg) is remarkably higher than that of R-pindolol (2.12 L/kg). Nevertheless, no significant differences in elimination rate constant and elimination half-life between both isomers were observed in pigs as the Cl_TOT_ of S-pindolol (161.26 ± 62.79 ml/min/kg) was significantly higher compared to R-pindolol (56.85 ± 17.46 ml/min/kg). The observed renal as well as the nonrenal clearance is significantly higher for S- than R-pindolol in pigs. In contrast, the net tubular secretion of R-pindolol and S-pindolol was not significantly different (p = 0.46). Conversely, in humans a stereoselective secretion of pindolol was present (Gross et al., 2001). The nonrenal clearances of both pindolol isomers observed in this study (157.98 & 55.51 ml/min/kg) were remarkably higher than the human values reported in literature (4.5–6.75 ml/min/kg) (Gross et al., 2001; Udy et al., 2014). Resulting from this high nonrenal clearance, a limited amount (2–3%) of unchanged pindolol isomers was recovered in urine. In other animal species pindolol seems also extensively metabolized resulting in rather small urinary excretion fraction of unchanged pindolol ranging between 0.6 and 4.3% for mouse, dog, and Rhesus monkey. With a value of 35%, the urinary excretion is remarkably higher in humans (Schwarz, 1982). This observation highlights the possible differences in metabolism between humans and pigs. Although some research is already performed concerning these differences, there is still a knowledge gap which emphasizes the need for studies dealing with pig–human differences in phase I and II biotransformation processes (Merrifield et al., 2011; Helke et al., 2016; Schelstraete et al., 2019). Furthermore, a marker that is only renally excreted for 2–% of the dose cannot be considered as a suitable marker for kidney function. Moreover, the limited renal excretion as a consequence of the high metabolism could be the reason for the high standard deviation observed for the secretion of the pindolol isomers, making evaluation of a stereoselective secretion difficult. It could be more appropriate to select a compound undergoing less metabolism to establish the cation secretion. An alternative marker could be famotidine (Dowling et al., 2001). However, just like for pindolol no porcine PK data of this compound is, to the author's knowledge, available in literature.

As for pindolol, no studies are available describing the use of fluconazole in pigs. Plasma protein binding of fluconazole in piglets was comparable (11%) compared to other species (~12%) like humans, dogs, rats, and mice (Humphrey et al., 1985). In addition, quite similar results in PK parameters were obtained (Ripa et al., 1993; Brammer and Coates, 1994; Gross et al., 2001). Nevertheless, the net tubular reabsorption of fluconazole in pigs was higher than that observed in humans. This difference was more pronounced when indexed for BW than when indexed for BSA. In this study, fluconazole has been used as a marker for the net tubular reabsorption, which takes both the active and passive reabsorption into account. However, fluconazole has been previously described by Tett et al. as an indicator for the passive tubular reabsorption (Tett et al., 2003). An important physiological variable which may affect this process is urinary pH. The urinary pH observed in the studied pigs was on average 5.53 ± 0.67, which is close to the average (pH = 6) observed in children (Liao and Churchill, 2001). As in humans, the renal clearance of fluconazole can be estimated over a 0–24 h interval, since no statistically significant difference was observed when compared to CL_R_ estimated over 48 h (Gross et al., 2001). This substantially simplifies the procedure and reduces the risk of loss of data due to leakages of the urine bags. Taking these considerations into account, fluconazole seems an appropriate marker to estimate the tubular reabsorption in pigs.

## Conclusion

In conclusion, iohexol, PAH, and fluconazole are suitable renal markers to assess the porcine renal function. On the other hand, pindolol is not a suitable renal marker due to the high nonrenal clearance in pigs compared to humans. This observation highlights the potential differences in metabolization capacity between human and pig. Generally, clearance values of humans and pigs correspond better when indexed to BSA than BW. The values of GFR, ERPF, anion secretion are within the same range for humans and pigs. Regarding the tubular reabsorption of fluconazole, slightly higher values were obtained for pigs. Nevertheless, these results indicate the pig could be an appropriate animal model to study renal drug elimination processes in humans.

## Data Availability Statement

The raw data supporting the conclusions of this article will be made available on reasonable request.

## Ethics Statement

The animal study was reviewed and approved by The Ethical Committee of the Faculty of Veterinary Medicine and the Faculty of Bioscience Engineering of Ghent University (EC2017/24).

## Author Contributions

LD, MD, SC, PDP, and PDC contributed to the conception and design of the study. LD performed and coordinated the animal trial, performed the bioanalytical, pharmacokinetic, and statistical analysis, and drafted the manuscript. MD aided in the pharmacokinetic analysis. SW, SP, and JR performed the bioanalytical analysis of pindolol and fluconazole. All authors contributed to the article and approved the submitted version.

## Funding

This study was supported by the Special Research Fund of Ghent University (grant number BOF16/DOC/285). JR would like to acknowledge funding from the Australian National Health and Medical Research Council for a Centre of Research Excellence (APP1099452) and a Practitioner Fellowship (APP1117065).

## Conflict of Interest

The authors declare that the research was conducted in the absence of any commercial or financial relationships that could be construed as a potential conflict of interest.
